# Correction: Ketamine/Xylazine-Induced Corneal Damage in Mice

**DOI:** 10.1371/journal.pone.0137559

**Published:** 2015-09-01

**Authors:** The PLOS ONE Staff

## Notice of Republication

This article was republished on August 20, 2015, to correct a paragraph of the Results section, which was mistakenly incorporated into the legend of Table 1. The publisher apologizes for the error. Please download this article again to view the correct version. The originally published, uncorrected article and the republished, corrected article are provided here for reference.

## Supporting Information

S1 File Originally published, uncorrected article. (PDF)

S2 File Republished, corrected article. (PDF)
