# Supplementary material for: Correction: Ketamine/Xylazine-Induced Corneal Damage in Mice
Source: PLoS One. 2015 Sep 1;10(9):e0137559. doi: 10.1371/journal.pone.0137559 (PMC4556641; doi:10.1371/journal.pone.0137559)
Supplement: S2 File — (PDF) [file pone.0137559.s002.pdf]

PLOS ONE

RESEARCH ARTICLE

Ketamine/Xylazine-Induced Corneal Damage in Mice

Demelza Koehn[1☯], Kacie J. Meyer[1☯], Nasreen A. Syed[2,3], Michael G. Anderson[1,2,4]*

1 Department of Molecular Physiology and Biophysics, University of Iowa, Iowa City, Iowa, United States of America, **2** Department of Ophthalmology and Visual Sciences, University of Iowa, Iowa City, Iowa, United States of America, **3** Department of Pathology, University of Iowa, Iowa City, Iowa, United States of America, **4** Center for the Prevention and Treatment of Visual Loss, Iowa City VA Health Care System, Iowa City, Iowa, United States of America

☯ These authors contributed equally to this work.
* michael-g-anderson@uiowa.edu

CrossMark
click for updates

OPEN ACCESS

Citation: Koehn D, Meyer KJ, Syed NA, Anderson MG (2015) Ketamine/Xylazine-Induced Corneal Damage in Mice. PLoS ONE 10(7): e0132804. doi:10.1371/journal.pone.0132804

Editor: Neena B. Haider, Schepens Eye Research Institute/Massachusetts Eye and Ear, Department of Ophthalmology, Harvard Medical School, Boston, MA, UNITED STATES

Received: July 8, 2014

Accepted: June 19, 2015

Published: July 29, 2015

Copyright: This is an open access article, free of all copyright, and may be freely reproduced, distributed, transmitted, modified, built upon, or otherwise used by anyone for any lawful purpose. The work is made available under the Creative Commons CC0 public domain dedication.

Data Availability Statement: All relevant data are within the paper.

Funding: This work was funded by the following: National Institutes of Health EY021436 (to DK) (http://www.nih.gov/) and National Institutes of Health EY017673 and EY018825 (to MGA) (http://www.nih.gov/). The funders had no role in study design, data collection and analysis, decision to publish, or preparation of the manuscript.

Competing Interests: Michael Anderson is a PLOS ONE Editorial Board member. This does not alter the

Abstract

Purpose

We have observed that the commonly used ketamine/xylazine anesthesia mix can induce a focally severe and permanent corneal opacity. The purpose of this study was to establish the clinical and histological features of this deleterious side effect, its sensitivity with respect to age and anesthesia protocol, and approaches for avoiding it.

Methods

Young C57BL/6J, C57BLKS/J, and SJL/J mice were treated with permutations of anesthesia protocols and compared using slit-lamp exams, optical coherence tomography, histologic analyses, and telemetric measurements of body temperature.

Results

Ketamine/xylazine induces corneal damage in mice with a variable frequency. Among 12 experimental cohorts, corneal damage associated with ketamine/xylazine was observed in 9 of them. Despite various treatments to avoid corneal dehydration during anesthesia, the frequency of corneas experiencing damage among responding cohorts was 42% (26% inclusive of all cohorts), which is significantly greater than the natural prevalence (5%). The damage was consistent with band keratopathy. It appeared as a white or gray horizontal band located proximal to the pupil and was positive for subepithelial calcium deposition with von Kossa stain.

Conclusions

The sum of our clinical and histological observations is consistent with ketamine/xylazine-induced band keratopathy in mice. This finding is relevant for mouse studies involving the eye and/or vision-dependent behavioral assays, which would both be prone to artifact

authors' adherence to PLOS ONE Editorial policies and criteria.

without appreciation of the damage caused by ketamine/xylazine anesthesia. Use of yohimbine is suggested as a practical means of avoiding this complication.

Introduction

Mice are widely utilized in many types of ophthalmic research and development[1–6], including the study of diseases of infancy and childhood that necessitate studies with young mice.[7–10] We have recently uncovered an unusual and deleterious influence that the commonly used ketamine/xylazine anesthesia mix has on the cornea of young mice. In searching the literature for a potential explanation of this phenomenon, we found that this complication is currently not widely appreciated. Here, we present data demonstrating that this anesthesia-induced side-effect is a form of band keratopathy and that it can readily be prevented by use of yohimbine.

Methods

Animals

The precise strain, age, and source, as well as treatments, of all mice in this study are summarized in Table 1. All animals were housed in non-barrier facilities maintained by the Office of Animal Resources at The University of Iowa. All animals were treated in accordance with the ARVO Statement for the Use of Animals in Ophthalmic and Vision Research. All experimental protocols were approved by the Animal Care and Use Committee of The University of Iowa.

Anesthesia and ocular examination

For all cohorts involving ketamine/xylazine, mice were injected with a standard mixture of ketamine/xylazine (intraperitoneal injection of 100 mg ketamine + 10 mg xylazine / kg body weight; Ketaset®, Fort Dodge Animal Health, Fort Dodge, IA; AnaSed®, Lloyd Laboratories, Shenandoah, IA). During induction of anesthesia, mice were placed in an empty cage and provided supplemental indirect warmth either by an electrical heating pad placed over the cage or an animal temperature controller connected to a rectal probe (TCAT-2LV controller, RET-3 probe, Physitemp Instruments, Clifton, NJ). Among cohorts receiving dilating drops (2% Cyclogyl, Alcon Laboratories, Fort Worth, TX), drops were applied immediately following anesthesia and after 2 minutes the corneas were then hydrated with balanced salt solution (BSS; Alcon Laboratories, Fort Worth, TX), Systane Ultra (Alcon Laboratories, Fort Worth, TX), GenTeal Mild to Moderate (Novartis Pharmaceuticals Corp., East Hanover, NJ), Hypo Tears (Novartis Pharmaceuticals Corp., East Hanover, NJ), Neomycin and Polymyxin B Sulfates and Bacitracin Zinc Ophthalmic Ointment USP (Bausch and Lomb, Rochester, NY), or Artificial Tears (AKORN Animal Health, Lake Forest, IL). Among cohorts receiving retinal scans by optical coherence tomography (OCT; Bioptigen, Morrisville, NC), the eye was exposed for 1 minute to OCT. During recovery from anesthesia, both eyes were hydrated as indicated in Table 1 and all mice were again placed in an empty cage and provided supplemental indirect warmth either by an electrical heating pad placed over the cage or an animal temperature controller connected to a rectal probe. A subset of mice was injected with yohimbine (intraperitoneal injection of 2 mg yohimbine / kg of body weight; Yobine®, Lloyd, Inc., Shenandoah, IA) upon placement in an empty cage for recovery. The incidence of corneal opacities was evaluated one week following anesthesia either by OCT or slit-lamp. Mice evaluated by OCT one week post-anesthesia were re-anesthetized, corneas were hydrated with BSS, and

Table 1. Summary of experiments.

Cohort	Experiment Date	Strain	Age	Sex	Source[a]	Anesthesia[b]	Drops and Hydration[c]	Yohimbine	OCT Retinal Scan[d]	Temperature Transponder	Supplemental Heat[e]	Eyes with damage
1	1/10/12	C57BL/6J	3 wks	F	JAX AX4	K/X	G + D		OU		Heating Pad	3/4 (75%)
	1/10/12	C57BL/6J	3 wks	F	JAX AX4	K/X	S + D		OU		Heating Pad	3/4 (75%)
	1/10/12	C57BL/6J	3 wks	F	JAX AX4	K/X	H + D		OU		Heating Pad	3/4 (75%)
	1/10/12	C57BL/6J	3 wks	F	JAX AX4	K/X	B + D		OS		Heating Pad	6/8 (75%)
	1/10/12	C57BL/6J	3 wks	F	JAX AX4	K/X	B + D		OU		Heating Pad	9/12 (75%)
2	1/24/12	C57BL/6J	12 wks	F	JAX AX4	K/X	B + D		OS		Heating Pad	2/8 (25%)
3	4/2/12	C57BL/6J	3 wks	F	JAX AX4	K/X	None				Heating Pad-cont.	0/4 (0%)
	4/2/12	C57BL/6J	3 wks	F	JAX AX4	K/X	None	Yes			Heating Pad-cont.	0/8 (0%)
	4/2/12	C57BL/6J	3 wks	F	JAX AX4	I	None				Heating Pad-cont.	0/8 (0%)
4	2/12/13	C57BL/6J	3 wks	F	JAX MP15	K/X	B		OU		Heating Pad	8/8 (100%)
	2/12/13	C57BL/6J	3 wks	F	JAX MP15	K/X	B	Yes	OU		Heating Pad	0/8 (0%)
	2/12/13	C57BL/6J	3 wks	F	JAX MP15	I	B		OU		Heating Pad	0/8 (0%)
5	8/8/13	C57BLKS/J	3 wks	F	IOWA	K/X	B			Yes	Heating Pad	2/4 (50%)
	8/8/13	C57BLKS/J	18 wks	F	IOWA	K/X	B			Yes	Heating Pad	0/4 (0%)
6	10/29/13	SJL/J	3 wks	F	IOWA	K/X	B				Heating Pad	4/10 (40%)
7	10/30/13	C57BLKS/J	3 wks	F	IOWA	K/X	B			Yes	Physitemp	2/6 (33.3%)
	10/30/13	C57BLKS/J	3 wks	F	IOWA	K/X	B			Yes	Physitemp-cont.	2/8 (25%)
8	3/6/14	C57BL/6J	3 wks	M/F	IOWA	K/X	B				Heating Pad	4/6 (66.7%)
	3/6/14	C57BL/6J	3 wks	M/F	IOWA	K/X	P-N				Heating Pad	0/8 (0%)
9	3/13/14	C57BL/6J	3 wks	F	IOWA	K/X	B				Heating Pad	1/4 (25%)
	3/13/14	C57BL/6J	3 wks	F	IOWA	K/X	P-AT				Heating Pad	0/6 (0%)
10	4/7/14	C57BL/6J	3 wks	F	JAX RB07	K/X	B				Heating Pad	0/10 (0%)
	4/7/14	C57BL/6J	3 wks	F	JAX RB07	K/X	P-AT				Heating Pad	0/10 (0%)
11	6/10/14	C57BL/6J	3 wks	F	JAX RB07	K/X	B				Heating Pad	7/14 (50%)
	6/10/14	C57BL/6J	3 wks	F	JAX RB07	K/X	P-N				Heating Pad	2/12 (14.3%)
	6/10/14	C57BL/6J	3 wks	F	JAX RB07	K/X	P-AT				Heating Pad	2/14 (16.7%)
12	6/10/14	SJL/J	3 wks	F	JAX RB04	K/X	B				Heating Pad	0/14 (0%)
	6/10/14	SJL/J	3 wks	F	JAX RB04	K/X	P-N				Heating Pad	0/12 (0%)
	6/10/14	SJL/J	3 wks	F	JAX RB04	K/X	P-AT				Heating Pad	0/14 (0%)

[a]JAX, received from The Jackson Laboratory, colony indicated, experiments initiated the day following receipt

[b]K/X, ketamine/xylazine; I, isoflurane

[c]G, Genteal; S, Systane; H, Hypotears; B, balanced salt solution; D, dilation drops; P-N, petrolatum with Neomycin antibiotic; P-AT, petrolatum-based artificial tears

[d]OU, both eyes; OS, left eye

[e]cont., continuous supplemental heat

doi:10.1371/journal.pone.0132804.t001

both corneas were imaged using OCT. To evaluate the natural prevalence of corneal opacities, slit-lamp examinations were performed on our colony of C57BL/6J mice (>1 year of age; $n = 72$ eyes). Histologic analyses followed previously described standard methodologies.[11] Among cohorts anesthetized with isoflurane, mice were anesthetized with 2.5% isoflurane + 100% oxygen for 15 minutes.

Telemetric monitoring of body temperature

Implantable temperature transponders (IPTT-300; Bio Medic Data Systems; Seaford, DE) were injected subcutaneously into 21-day-old ($n = 3$) and 18-week-old ($n = 3$) C57BLKS/J mice anesthetized with isoflurane. Two days post-injection, body temperature was measured using an external recording device (DAS-7009 small smart reader; Bio Medic Data Systems; Seaford, DE) and then mice were injected with ketamine/xylazine, placed in an empty cage, and provided supplemental indirect warmth by an electrical heating pad placed over the cage.

Immediately upon anesthesia, temperature was recorded (t = 0) and mice were removed from heat and placed on the OCT platform for 2.5 minutes. Mice were then given standard indirect warmth by an electrical heating pad placed over the cage for the recovery period and body temperature was monitored throughout recovery for up to 40 minutes. Body temperature measurements were taken every 2 minutes for the first 10 minutes, and then every 5 minutes for the remainder of the experiment.

Results

We initially observed an induction of central corneal opacities in longitudinal studies of postnatal ocular maturation in mice using OCT. For the original study, anesthetized mice were to be imaged on a weekly basis starting at 3 weeks of age. Based on prior studies of ocular growth in C57BL/6J mice, we expected to observe a clear and healthy cornea that gradually thickened through subsequent weeks of postnatal development.[12, 13] Unexpectedly, at the second week of imaging most of the corneas were damaged with opacities (Fig 1), making our original planned studies impossible. Subsequent characterization identified several features consistent with band keratopathy.[14, 15] Observed by OCT and slit-lamp examination, the opacities were centrally located, appearing as a horizontal band between the subepithelial layer and anterior stroma (Fig 1). Eyes appeared free from inflammation and there was no evidence of vascularization. Histologically, damaged corneas showed accumulation of a granular substance that raised the epithelial layer (Fig 2). Von Kossa staining showed the appearance of brown-black granules at the site of the lesion, indicating calcium deposition characteristic of band keratopathy (Fig 3). To our knowledge, neither band keratopathy nor corneal damage of any type

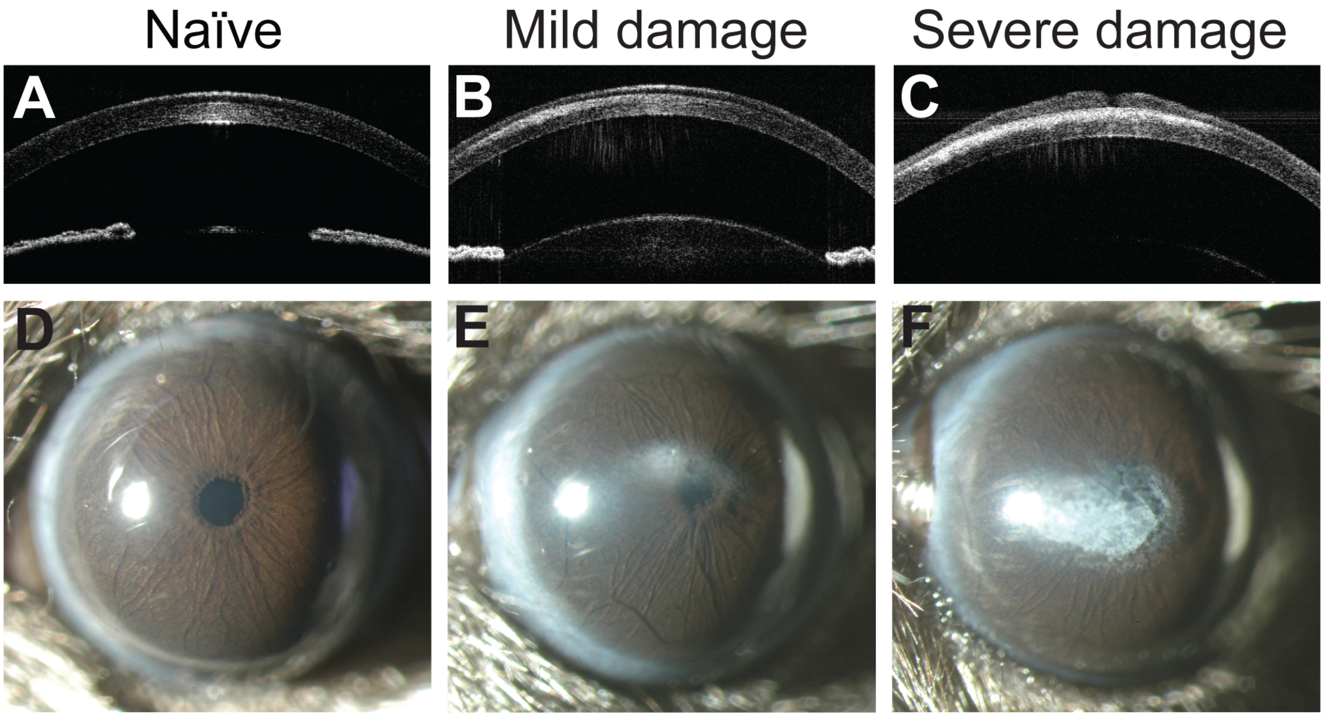

Fig 1. Gross morphological changes in four-week-old C57BL/6J corneas after exposure to ketamine/xylazine. Optical coherence tomography (OCT) of the anterior segment (**A-C**) and slit-lamp images (**D-F**) of mice injected with ketamine/xylazine show a range of mild (*middle column*) to severe (*right column*) corneal damage. In the OCT images, note the presence of corneal opacities between the stromal and epithelial layers. Naïve C57BL/6J mice (no prior injection with ketamine/xylazine; *left column*) have healthy, clear corneas and are free of opacity.

doi:10.1371/journal.pone.0132804.g001

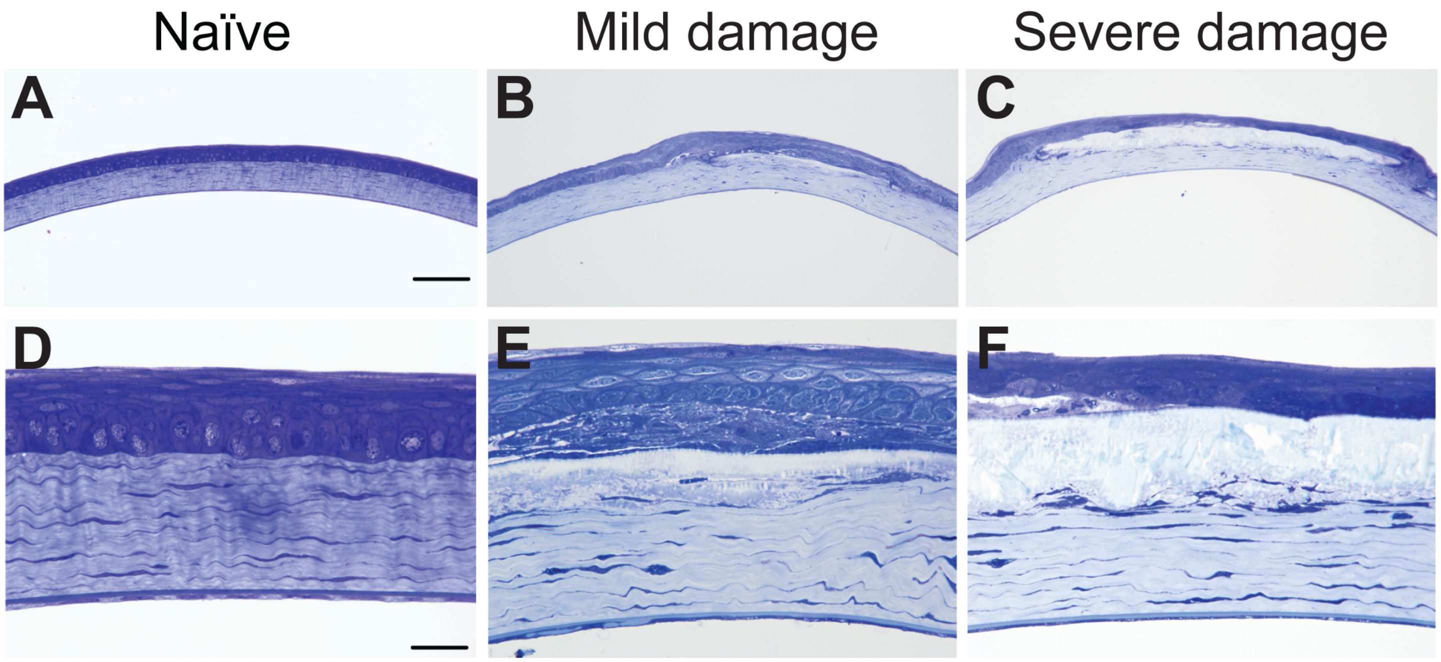

Fig 2. Histological changes in four-week-old C57BL/6J corneas after exposure to ketamine/xylazine. Glutaraldehyde-fixed, plastic-embedded sections stained with toluidine blue at low (**A-C**) and high (**D-F**) magnification. Naïve C57BL/6J corneas (*left column*) showed normal epithelial cell and stromal morphologies. Corneas with mild damage (*middle column*) and severe damage (*right column*) showed deposition of a crystalline substance between the epithelial and stromal layers. There was a loss of cellularity of the basal and wing epithelial cells and flattening of the stromal lamellae compared to corneas of naïve mice. Scale bars: A-C, 50 μm; D-F, 10 μm.

doi:10.1371/journal.pone.0132804.g002

has previously been reported in association with this strain of mice, imaging modality, or anesthesia protocol.

To suggest factors influencing the induction of these opacities, we next performed a series of experiments testing the effects of various permutations to each component of the experiment (Table 1). Among three-week-old mice receiving ketamine/xylazine anesthesia and standard post-anesthetic follow-up (supplemental warmth during induction and recovery, treatments to

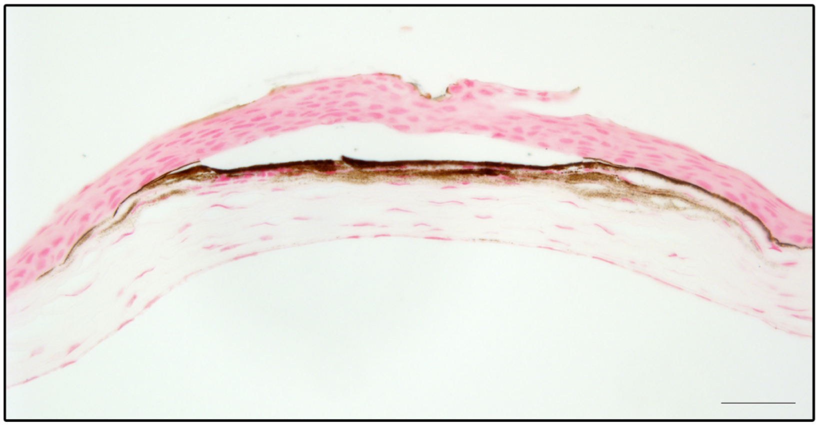

Fig 3. Calcium deposition of damaged corneas. Paraffin-embedded sections were stained with von Kossa. Calcium deposition is indicated by the brown staining at the interface of the epithelial and stromal layers. Scale bar, 20 μm.

doi:10.1371/journal.pone.0132804.g003

prevent corneal dehydration), 26% of all eyes exhibited corneal damage after 1 week (n = 55/210 eyes). Opacities were induced in cohorts of mice newly received from The Jackson Laboratory and cohorts born from existing colonies at The University of Iowa. Thus, the opacities were not obviously related to husbandry. Corneal opacities were observed in C57BL/6J, C57BLKS/J and SJL/J mice, inbred strains of mice with disparate central corneal thicknesses.[11] Thus, the induced opacities were also not a phenomenon unique to a single strain of mice. Opacities were equally present regardless of whether or not an eye had previously been imaged by OCT. Thus, the opacities were not the consequence of the imaging modality. Dilating drops were ruled out as a causative factor because their omission did not prevent the corneal damage. Having eliminated the above components as potential causative factors, the remaining agent utilized with all of the mice with corneal damage was ketamine/xylazine.

To explore the potentially damaging influence of ketamine/xylazine, we repeated the experiment with a cohort of 3-week-old C57BL/6J mice using isoflurane as a substitute anesthetic, yohimbine (Fig 4), a xylazine antagonist that accelerates recovery from ketamine/xylazine anesthesia, or using ketamine/xylazine with only standard post-anesthetic follow-up as a control (Table 1; cohort 4). No corneal damage was observed in any of the eyes from mice with either treatment (n = 0/8 eyes per treatment), whereas the control mice for this cohort had damage in all eyes (n = 8/8). Combined, these data support ketamine/xylazine as the damage-inducing agent.

To test the hypothesis that ketamine/xylazine anesthesia induces corneal opacities in mice, we compared the incidence in cohorts of naïve mice versus those that had been anesthetized with ketamine/xylazine. Corneal opacities are not a known naturally occurring feature of C57BL/6J, C57BLKS/J, or SJL/J mice and we have previously reported detecting only a single instance of a corneal opacity among a screen for corneal phenotypes among these strains.[11] To confirm the rate of naturally occurring central corneal opacities analogous to those we observed in our studies, we screened a cohort of naïve C57BL/6J mice (>1 year old) from our colony with a slit-lamp and observed this type of central corneal opacity to occur at a frequency of 5% (n = 4/72 eyes), which is significantly less than the frequency among ketamine/xylazine-treated responding cohorts (n = 60/144 eyes; 41.7%; $p < 0.0001$, Chi Square test) or all ketamine/xylazine-treated mice (n = 60/224 eyes; 26.8%; $p < 0.0001$, Chi Square test).

In considering potential mechanisms contributing to this phenomenon, we hypothesized that body temperature might have a role. Ketamine/xylazine anesthesia is known to cause a decrease in body temperature[16], as confirmed by our measurements using subcutaneous transponders throughout anesthesia in young and adult mice (Fig 5). To test whether this drop in body temperature contributes to the corneal damage, 3-week-old mice were anesthetized with ketamine/xylazine (without yohimbine), but rather than transferring the mice to the OCT for imaging, were instead maintained constantly with externally supplied warmth (delivered either by use of an electric heating pad or an animal temperature controller connected to a rectal probe) during all stages of the experiment. After one week, 17% (n = 2/12 eyes) of the mice developed opacities (Cohorts 3 & 7, Table 1). Thus, body temperature fluctuations may influence the corneal damage, but our attempts to minimize changes in body temperature were none-the-less unsuccessful in completely preventing it.

We also considered corneal desiccation as a potential mechanism. Opacities were observed in mice receiving independent water-based solutions influencing corneal dehydration (Systane Ultra, Alcon Laboratories, Fort Worth, TX; GenTeal Mild to Moderate, Novartis Pharmaceuticals Corp., East Hanover, NJ; Hypo Tears, Novartis Pharmaceuticals Corp., East Hanover, NJ). Use of petrolatum-based ointments (Artificial Tears, AKORN Animal Health, Lake Forest, IL; Neomycin and Polymyxin B Sulfates and Bacitracin Zinc Ophthalmic Ointment USP, Bausch

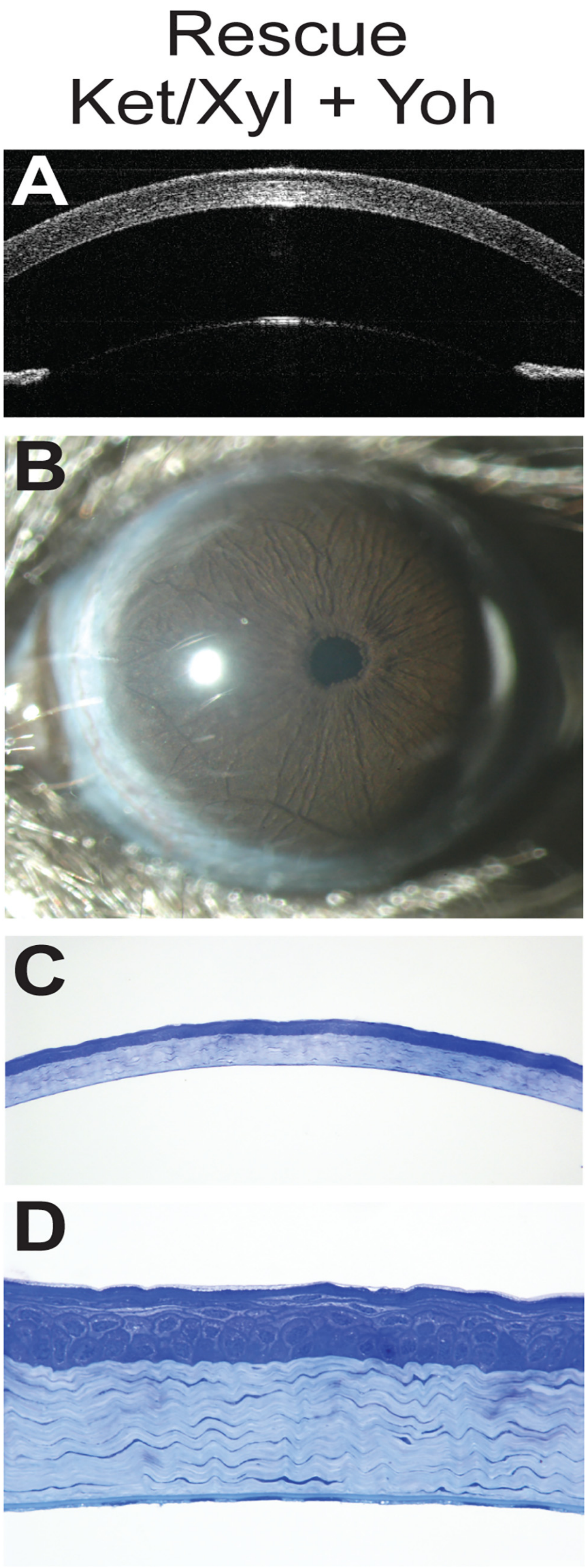
Rescue
Ket/Xyl + Yoh
A
B
C
D

Fig 4. Rescue of ketamine/xylazine-induced corneal damage by yohimbine. OCT (**A**) and slit-lamp (**B**) images show mice that were injected with yohimbine as a post-anesthesia follow-up are clear from opacity. The corneal epithelial cell layers and the stromal lamellae (**C, D**) look normal in yohimbine-injected mice. Scale bars: C, 50 μm; D, 10 μm.

doi:10.1371/journal.pone.0132804.g004

and Lomb, Rochester, NY;), however, potentially influences the incidence of corneal opacities, but was not able to completely prevent corneal damage.

Discussion

Ketamine/xylazine is one of the most common anesthetics utilized with mice,[17] but to our knowledge, a corneal-damaging influence of ketamine/xylazine has not previously been reported for mice. If not prevented or controlled for, this phenomenon could obviously have a significant impact on many different kinds of experiments, altering the anatomy and physiology of the anterior chamber and distorting the ability to image or record responses from the entire eye. Because this phenomenon is of broad experimental significance to ophthalmic research using mice, we felt it prudent to bring attention to the issue despite some lingering questions concerning the mechanism.

In investigating the literature, there are several potential side-effects of ketamine/xylazine. It is known that ketamine/xylazine can induce reversible cataracts in rodents[18] and can affect intraocular pressure.[19, 20] Corneal damage in young rats due to ketamine/xylazine has previously been observed. In 1988, it was reported by Guillet and colleagues that 84% of 3-week-old rats anesthetized with ketamine/xylazine developed corneal lesions.[21] No lesions were observed in rats administered ketamine or xylazine alone, nor if rats were treated with

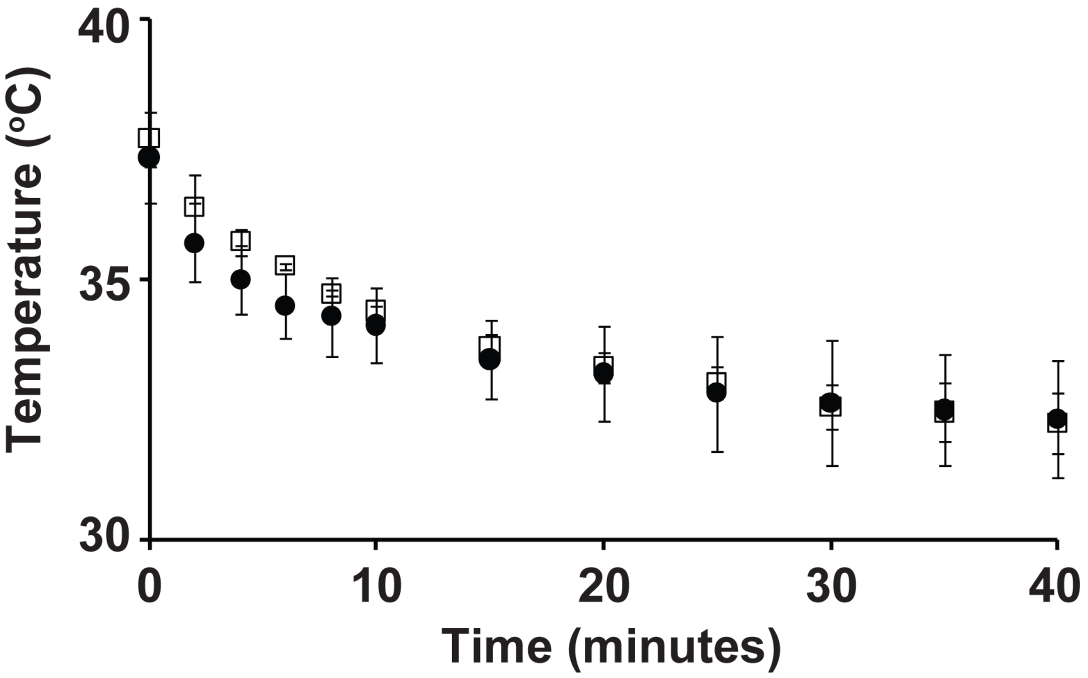

Fig 5. Body temperature loss during ketamine/xylazine anesthesia of 3-week-old (*filled circles*) versus 3-month-old (*open squares*) C57BLKS/J mice. Immediately upon anesthesia, mice were placed on the OCT platform for 2.5 minutes and then given indirect heat for the remainder of the experiment. There is no significant difference ($P > 0.05$ at all time points, unpaired t-tests) in body temperature loss between young (small) and adult (large) mice ($n = 3$ mice per group). Error bars are standard deviation.

doi:10.1371/journal.pone.0132804.g005

yohimbine during recovery. Similar results in rats were subsequently reported by Tita et al.[22] and Turner and Albassam who additionally showed that if rats were anesthetized with isoflurane instead of ketamine/xylazine, damage was avoided completely.[23] Similar to our observations, these studies using rats injected with ketamine/xylazine reported damage involving central corneal opacities and seem likely to indicate that the same deleterious side-effect we have observed occurs in multiple rodent species.

The mechanism for this phenomenon is unknown. In both humans and animals, band keratopathy has been associated with a variety of ocular and systemic conditions that, through a variety of mechanisms, leave Bowman's membrane prone to calcification in areas of greatest environmental exposure.[24, 25] In the context of anterior uveitis, band keratopathy is more frequently observed in children than adults.[26, 27] Current hypotheses of pathophysiology center on events such as tear evaporation and changes in pH that may promote calcium precipitation, but much remains unknown concerning the precise mechanism(s).[25] Our studies of the ketamine/xylazine-induced form of this disease are consistent with a complex age-dependent interaction involving combined use of both compounds, though other mechanisms can't be ruled out. Ketamine is primarily classified as an NMDA receptor antagonist, although interactions with other receptors and channels have also been reported.[28] Xylazine is an α2 adrenergic agonist and is commonly used with ketamine as an adjunct agent because of its analgesic and muscle relaxant properties.[29] Drawing from the experiments of Guillet et al. who showed that the damage in rats does not occur with ketamine or xylazine alone, and that the damage can be prevented by treatment with yohimbine (an α2 adrenergic antagonist), it appears that the phenomenon may be due to a complex drug interaction or physiologic consequence of the ketamine/xylazine mixture.Potential implications of this observation to humans are unclear. Ketamine is used clinically as a pediatric anesthetic[30] and increasingly as an antidepressant.[31] The safety of ketamine use in children has had some controversy.[32, 33] However, we are unaware of any reports of corneal damage in children and there is only one case report linking ketamine use to corneal damage in an adult.[34] Ketamine is also used non-clinically as a recreational drug.[35] To our knowledge, the use of xylazine is limited to veterinary practice. It is unknown, but possible, that other α2 adrenergic agonists that are used in human clinical practice could have a similar interaction if used concurrently with ketamine. Because reports of irreversible ketamine/xylazine-induced corneal damage are thus far limited to rodents, and the combination of scenarios placing humans potentially at risk unlikely, a case for imminent human relevance seems challenged but not impossible.

Among caveats of this study, an issue regarding variability merits particular mention. Cohort-to-cohort variability was notably present in our study. Among 12 total experimental cohorts comprising all of the experiments described above (see Table 1), induced corneal damage was not observed in controls (i.e., mice injected with ketamine/xylazine, given BSS drops to maintain corneal hydration, and provided standard post-anesthetic care) for 3 of them (Cohorts 3, 10, 12; yielding a frequency for ketamine/xylazine-induced damage of 42% for corneas within responding cohorts and 26% inclusive of all cohorts). Although we were unable to isolate a factor explaining this variability, it remains possible that a nuance of procedure, environment, or physiology may in fact explain this phenomenon.

It is important to draw a distinction between statistically supported conclusions of our study (i.e., ketamine/xylazine increases the incidence of band keratopathy in mice) versus several observations that do not rigorously distinguish various mechanisms. As discussed above, the cohort-to-cohort variability makes this phenotype difficult to study. Regarding incidence, based on our past experiences using ketamine/xylazine with mice[11, 36, 37], we anecdotally have the impression that damage is more likely to occur in young mice (< 1 mo of age), but our current study was not sufficiently powered to test this robustly. With respect to

mechanism, it's important to point out that our current results do not distinguish whether this occurs due primarily to a drug interaction (ketamine, xylazine, or their combined use), or a secondary physiological consequence such as decreased body temperature, corneal desiccation, or time under anesthesia. Regarding prevention, although we observed indications that petrolatum-based ointment and careful monitoring of body temperature may reduce the incidence of corneal opacities, we have added yohimbine to our standard anesthesia protocol because it was the most effective and efficient solution in our hands.

In sum, we have observed that ketamine/xylazine anesthesia induces corneal damage in mice. A small body of literature from experiments in rats might have predicted this phenomenon, but our unfamiliarity with it–perhaps the unfamiliarity of most in our field–caused an experimental complication with our intended experiments. The precise age-dependency, strain-dependency, and mechanism of this phenomenon remain ill-defined, so we encourage investigators to examine its influence more closely in their own experiments.

Author Contributions

Conceived and designed the experiments: DK KJM MGA. Performed the experiments: DK KJM NAS. Analyzed the data: DK KJM NAS MGA. Contributed reagents/materials/analysis tools: NAS MGA. Wrote the paper: DK KJM MGA.

References

1. Bartsch U, Galliciotti G, Jofre GF, Jankowiak W, Hagel C, Braulke T. Apoptotic photoreceptor loss and altered expression of lysosomal proteins in the nclf mouse model of neuronal ceroid lipofuscinosis. Invest Ophthalmol Vis Sci. 2013; 54(10):6952–9. doi: 10.1167/iovs.13-12945 PMID: 24084090.
2. Svenson KL, Gatti DM, Valdar W, Welsh CE, Cheng R, Chesler EJ, et al. High-resolution genetic mapping using the Mouse Diversity outbred population. Genetics. 2012; 190(2):437–47. doi: 10.1534/genetics.111.132597 PMID: 22345611; PubMed Central PMCID: PMC3276626.
3. Dursun I, Jakubowska-Dogru E, van der List D, Liets LC, Coombs JL, Berman RF. Effects of early postnatal exposure to ethanol on retinal ganglion cell morphology and numbers of neurons in the dorsolateral geniculate in mice. Alcoholism, clinical and experimental research. 2011; 35(11):2063–74. doi: 10.1111/j.1530-0277.2011.01557.x PMID: 21651582; PubMed Central PMCID: PMC3410545.
4. Mao M, Hedberg-Buenz A, Koehn D, John SW, Anderson MG. Anterior segment dysgenesis and early-onset glaucoma in nee mice with mutation of Sh3pxd2b. Invest Ophthalmol Vis Sci. 2011; 52(5):2679–88. doi: 10.1167/iovs.10-5993 PMID: 21282566; PubMed Central PMCID: PMC3088558.
5. Seo S, Mullins RF, Dumitrescu AV, Bhattarai S, Gratie D, Wang K, et al. Subretinal gene therapy of mice with Bardet-Biedl syndrome type 1. Invest Ophthalmol Vis Sci. 2013; 54(9):6118–32. doi: 10.1167/iovs.13-11673 PMID: 23900607; PubMed Central PMCID: PMC3771708.
6. Son AI, Cooper MA, Sheleg M, Sun Y, Kleiman NJ, Zhou R. Further analysis of the lens of ephrin-A5-/- mice: development of postnatal defects. Molecular vision. 2013; 19:254–66. PMID: 23401654; PubMed Central PMCID: PMC3566898.
7. Graw J. Mouse models of cataract. Journal of genetics. 2009; 88(4):469–86. PMID: 20090208.
8. Islam MS. Animal models of diabetic neuropathy: progress since 1960s. Journal of diabetes research. 2013; 2013:149452. doi: 10.1155/2013/149452 PMID: 23984428; PubMed Central PMCID: PMC3745837.
9. McKinnon SJ, Schlamp CL, Nickells RW. Mouse models of retinal ganglion cell death and glaucoma. Experimental eye research. 2009; 88(4):816–24. doi: 10.1016/j.exer.2008.12.002 PMID: 19105954; PubMed Central PMCID: PMC3056071.
10. Pennesi ME, Neuringer M, Courtney RJ. Animal models of age related macular degeneration. Molecular aspects of medicine. 2012; 33(4):487–509. doi: 10.1016/j.mam.2012.06.003 PMID: 22705444; PubMed Central PMCID: PMC3770531.
11. Lively GD, Jiang B, Hedberg-Buenz A, Chang B, Petersen GE, Wang K, et al. Genetic dependence of central corneal thickness among inbred strains of mice. Investigative ophthalmology & visual science. 2010; 51(1):160–71. Epub 2009/08/28. doi: 10.1167/iovs.09-3429 PMID: 19710407; PubMed Central PMCID: PMC2869057.

12. Song J, Lee YG, Houston J, Petroll WM, Chakravarti S, Cavanagh HD, et al. Neonatal corneal stromal development in the normal and lumican-deficient mouse. Invest Ophthalmol Vis Sci. 2003; 44(2):548–57. PMID: 12556382; PubMed Central PMCID: PMC1853375.
13. Zieske JD. Corneal development associated with eyelid opening. The International journal of developmental biology. 2004; 48(8–9):903–11. doi: 10.1387/ijdb.041860jz PMID: 15558481.
14. Eagle RC. Eye pathology: an atlas and text, 2nd Ed. Philadelphia, PA: Lippincott Williams and Wilkins; 2011.
15. Fario QA, Sugar A. Conjunctival and corneal degenerations. In: Yanoff M, Duker JS, editors. Ophthalmology, 2nd Ed. St. Louis, MO: Mosby; 2004.
16. Gaertner DJ, Hallman TM, Hankenson FC, Batchelder MA. Anesthesia and analgesia for laboratory rodents. In: Fish RE, Brown MJ, Danneman PJ, Karas AZ, editors. Anesthesia and analgesia in laboratory animals. San Diego, CA: Academic Press; 2008.
17. Stokes EL, Flecknell PA, Richardson CA. Reported analgesic and anaesthetic administration to rodents undergoing experimental surgical procedures. Lab Anim. 2009; 43(2):149–54. Epub 2009/01/01. doi: 10.1258/la.2008.008020 PMID: 19116297.
18. Calderone L, Grimes P, Shalev M. Acute reversible cataract induced by xylazine and by ketamine-xylazine anesthesia in rats and mice. Experimental eye research. 1986; 42(4):331–7. PMID: 3754819.
19. Ding C, Wang P, Tian N. Effect of general anesthetics on IOP in elevated IOP mouse model. Experimental eye research. 2011; 92(6):512–20. doi: 10.1016/j.exer.2011.03.016 PMID: 21457709; PubMed Central PMCID: PMC3116023.
20. Qiu Y, Yang H, Lei B. Effects of Three Commonly Used Anesthetics on Intraocular Pressure in Mouse. Curr Eye Res. 2013. doi: 10.3109/02713683.2013.845224 PMID: 24215504.
21. Guillet R, Wyatt J, Baggs RB, Kellogg CK. Anesthetic-induced corneal lesions in developmentally sensitive rats. Invest Ophthalmol Vis Sci. 1988; 29(6):949–54. Epub 1988/06/01. PMID: 3372167.
22. Tita B, Leone MG, Casini ML, Corubolo C, Bordi F, Guidolin D, et al. Corneal toxicity of xylazine and clonidine, in combination with ketamine, in the rat. Ophthalmic Res. 2001; 33(6):345–52. Epub 2001/11/27. PMID: 11721188.
23. Turner PV, Albassam MA. Susceptibility of rats to corneal lesions after injectable anesthesia. Comp Med. 2005; 55(2):175–82. Epub 2005/05/12. PMID: 15884781.
24. Hoffman RW, Yang HK, Waggie KS, Durham JB, Burge JR, Walker SE. Band keratopathy in MRL/l and MRL/n mice. Arthritis and rheumatism. 1983; 26(5):645–52. PMID: 6847726.
25. Jhanji V, Rapuano CJ, Vajpayee RB. Corneal calcific band keratopathy. Current opinion in ophthalmology. 2011; 22(4):283–9. doi: 10.1097/ICU.0b013e3283477d36 PMID: 21537183.
26. Jones NP. Uveitis, 2nd Ed. London, UK: JP Medical Ltd.; 2013.
27. O'Connor GR. Calcific band keratopathy. Transactions of the American Ophthalmological Society. 1972; 70:58–81. PMID: 4677022; PubMed Central PMCID: PMC1310442.
28. Sinner B, Graf BM. Ketamine. Handb Exp Pharmacol. 2008;(182:):313–33. Epub 2008/01/05. doi: 10.1007/978-3-540-74806-9_15 PMID: 18175098.
29. Greene SA, Thurmon JC. Xylazine—a review of its pharmacology and use in veterinary medicine. J Vet Pharmacol Ther. 1988; 11(4):295–313. Epub 1988/12/01. PMID: 3062194.
30. Bergman SA. Ketamine: review of its pharmacology and its use in pediatric anesthesia. Anesth Prog. 1999; 46(1):10–20. Epub 1999/11/07. PMID: 10551055; PubMed Central PMCID: PMC2148883.
31. Skolnick P, Popik P, Trullas R. Glutamate-based antidepressants: 20 years on. Trends Pharmacol Sci. 2009; 30(11):563–9. Epub 2009/10/20. doi: 10.1016/j.tips.2009.09.002 PMID: 19837463.
32. Bhutta AT. Ketamine: a controversial drug for neonates. Semin Perinatol. 2007; 31(5):303–8. Epub 2007/10/02. doi: 10.1053/j.semperi.2007.07.005 PMID: 17905185.
33. Dolansky G, Shah A, Mosdossy G, Rieder M. What is the evidence for the safety and efficacy of using ketamine in children? Paediatr Child Health. 2008; 13(4):307–8. Epub 2009/04/02. PMID: 19337601; PubMed Central PMCID: PMC2529431.
34. Starte JM, Fung AT, Kerdraon YA. Ketamine-associated corneal edema. Cornea. 2012; 31(5):572–4. Epub 2012/02/16. doi: 10.1097/ICO.0b013e31823f8af4 PMID: 22333663.
35. Wood D, Cottrell A, Baker SC, Southgate J, Harris M, Fulford S, et al. Recreational ketamine: from pleasure to pain. BJU Int. 2011; 107(12):1881–4. Epub 2011/02/15. doi: 10.1111/j.1464-410X.2010.10031.x PMID: 21314885.
36. Lively GD, Koehn D, Hedberg-Buenz A, Wang K, Anderson MG. Quantitative trait loci associated with murine central corneal thickness. Physiological genomics. 2010; 42(2):281–6. doi: 10.1152/physiolgenomics.00140.2009 PMID: 20423963; PubMed Central PMCID: PMC3032283.

37. Anderson MG, Libby RT, Mao M, Cosma IM, Wilson LA, Smith RS, et al. Genetic context determines susceptibility to intraocular pressure elevation in a mouse pigmentary glaucoma. BMC biology. 2006; 4:20. Epub 2006/07/11. doi: 10.1186/1741-7007-4-20 PMID: 16827931; PubMed Central PMCID: PMC1543659.
